# Can multiparametric FDG-PET/MRI analysis really enhance the prediction of myocardial recovery after CTO revascularization? A machine learning study

**DOI:** 10.1016/j.zemedi.2025.03.003

**Published:** 2025-04-23

**Authors:** Alberto Villagran Asiares, Teresa Vitadello, Osvaldo M. Velarde, Sylvia Schachoff, Tareq Ibrahim, Stephan G. Nekolla

**Affiliations:** a Nuklearmedizinische Klinik und Poliklinik, TUM Klinikum, Klinikum rechts der Isar, School of Medicine and Health, Technical University of Munich, Munich, Germany; b Klinik und Poliklinik für Innere Medizin I, TUM Klinikum, Klinikum rechts der Isar, School of Medicine and Health, Technical University of Munich, Munich, Germany; c Deutsches Zentrum für Herz-Kreislauf-Forschung e.V., partner site Munich Heart Alliance, Munich, Germany; d Biomedical Engineering Department, The City College of New York, New York, NY 10030, United States; e Klinik und Poliklinik für Kinder- und Jugendpsychiatrie, Psychosomatik und Psychotherapie. Klinikum der Ludwig-Maximilians-Universität München, Munich, Germany

**Keywords:** Cardiac imaging, Hybrid positron emission tomography/magnetic resonance imaging, Machine Learning, Revascularization, Myocardial viability assessment, Wall motion recovery prediction

## Abstract

**Purpose:**

To comprehensively evaluate the effectiveness of FDG-PET/MRI multiparametric analysis in predicting myocardial wall motion recovery following revascularization of chronic coronary total occlusions (CTO), incorporating both traditional and machine learning approaches.

**Methods:**

This retrospective study assessed fluorine-18 fluorodeoxyglucose uptake (FDG), late gadolinium enhanced MR imaging (LGE), and MR wall motion abnormalities (WMA) of the left ventricle walls of a clinical cohort with 21 CTO patients (62 ± 9 years, 20 men). All patients were examined using a PET/MRI prior to revascularization and a follow-up cardiac MRI six months later. Prediction models for wall motion recovery after perfusion restoration were developed using linear and nonlinear algorithms as well as multiparametric variables. Performance and prediction explainability were evaluated in a 5x2 cross-validation framework, using ROC AUC and McNemar’s test modified for clustered matched-pair data, and Shapley values.

**Results:**

Based on 79 CTO-subtended myocardial wall segments with wall motion abnormalities at baseline, the reference logistic regression model LGE + FDG obtained 0.55(SE = 0.07) in the clustered ROC AUC (cROC AUC) and 0.17(0.05) in the Global Absolute Shapley value. The reference outperformed FDG standalone in cROC AUC (-35(17) %, p < 0.0001), but not LGE standalone (11(12) %, p > 0.05). There were no statistically significant differences between the marginal probabilities of success of these three models. Moreover, no significant improvements (differences < 10 % in cROC AUC, and < 20 % in Global Absolute Shapley, p > 0.05) were found when using mixed effects logistic regression, decision tree, k-nearest neighbor, Naive Bayes, random forest, and support vector machine, with multiparametric combinations of FDG, LGE, and/or WMA.

**Conclusion:**

In this clinical cohort, adding more complex interactions between PET/MRI imaging of cardiac function, infarct extension, and/or metabolism did not enhance the prediction of wall motion recovery after perfusion restoration. This finding raises the question whether multiparametric FDG-PET/MRI analysis has demonstrable benefits in risk stratification for CTO revascularization. Further studies with larger cohorts and external validation datasets are crucial to clarify this question and refine the role of multiparametric imaging in this context.

## Highlights

• Complex procedures such as CTO revascularization demand reliable prediction of myocardial recovery in the decision-making.
• Machine learning-based multiparametric FDG-PET/MRI analysis did not improve the prediction of wall motion recovery after PCI revascularization in a clinical CTO cohort.
• The benefits of multiparametric FDG-PET/MRI analysis in this field remain debatable, regardless of the employment of machine learning algorithms.

## Graphical abstract

**Figure:**
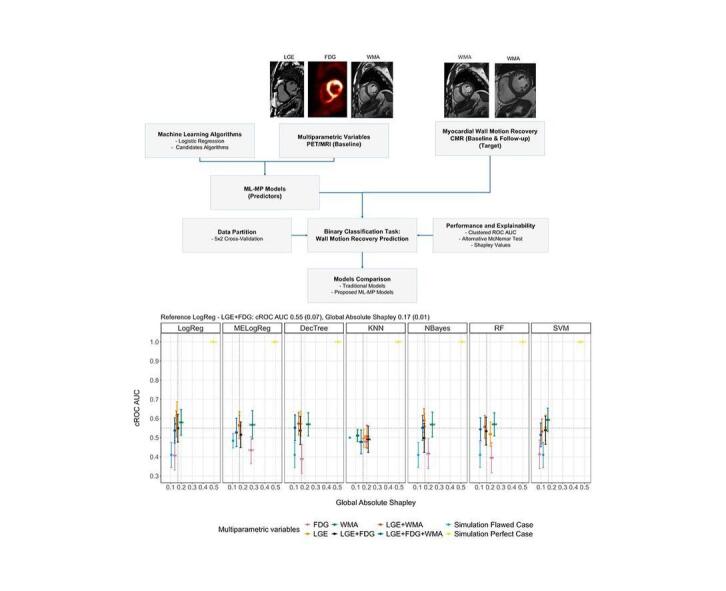

## Introduction

The coronary perfusion in patients with chronic total occlusion (CTO) can be restored via a percutaneous coronary intervention (PCI); a surgical procedure that exhibits high complication rates, such as perforation, coronary dissection, major bleeding, myocardial infarction, and death [[1], [2], [3]]. Pre-PCI myocardial viability assessments have been considered in the decision-making process using diverse cardiac imaging modalities [[4], [5], [6], [7]]. As such, viable myocardial segments with wall motion abnormalities (WMA) at baseline are supposed to show wall motion recovery after perfusion restoration (the viability hypothesis) [[7], [8], [9]].

In this context, previous studies recommend a multimodal PET/MRI analysis for a viability assessment exam in CTO patients [10,11]. They found that the linear combination of metabolism (from PET-FDG, fluorine-18 fluorodeoxyglucose) and infarct extension (from MRI-LGE, late gadolinium enhanced imaging) enhances the prediction of wall motion recovery post-revascularization in small-scale CTO cohorts, in comparison to single-modality approaches. Nevertheless, the performances of the predictor models were exclusively estimated on the same dataset they were trained on, as opposed to an unseen dataset. This implies a lack of generalizability estimation with the risk of high performance bias, which undermines of the clinical utility of these results [12,13]. It is essential to further investigate the benefits of multiparametric PET/MRI analysis in predicting wall motion recovery using comprehensive prediction workflows [14].

Additionally, machine learning algorithms have shown promise in identifying intricate interactions beyond linear combinations [[15], [16], [17]]. This, in turn, may lead to enhanced performance in classification tasks such as myocardial infarction detection, risk of arrhythmias, and diagnostic differentiation [[18], [19], [20], [21], [22]]. Moreover, apart from metabolism and infarct extension, findings of previous echocardiography-based studies highlighted benefits of including WMA in patient stratification for revascularization and cardiac events prognosis [[23], [24], [25]]. Consequently, the combination of multimodal multiparametric models and machine learning classifiers has the potential to improve the prediction of the myocardial wall motion recovery.

The objective of this study was to comprehensively evaluate the ability of FDG-PET/MRI multiparametric models in predicting myocardial wall motion recovery after revascularization. First, the performance robustness of previously studied predictor models was assessed in a cross-validation approach. Subsequently, a comparative analysis was performed between the previously recommended linear FDG-PET/MRI model and novel machine learning-boosted multiparametric models (ML-MP models). The prediction workflow addressed generalizability estimation, model explainability, intra-subject correlations, and sampling-derived bias in a retrospective clinical CTO cohort. We hypothesize that ML-MP models robustly enhance the prediction of myocardial wall motion recovery after a CTO revascularization.

## Material and methods

### Patient cohort

This retrospective work was conducted on a longitudinal CTO cohort previously described [11]. It consisted of 22 patients with symptomatic coronary artery disease, chronic total occlusion of a coronary artery, and evidence of wall motion abnormalities. Cardiac imaging was performed prior to PCI (baseline) and six months after (follow-up) revascularization. The inclusion criteria for this work were the presence of wall motion abnormalities at baseline in the corresponding CTO coronary artery treated (CTO-subtended artery), and complete PET and MRI information available (no missing values). All subjects provided their written informed consent in accordance with the guidelines of the local ethics board that approved the study.

### Imaging acquisition

Cardiac imaging was conducted using an ECG-gated hybrid PET/MRI system (Biograph mMR, Siemens Healthcare, Erlangen, Germany). A PET/MRI viability exam was performed at baseline before revascularization, followed by a cardiac MR (CMR) for therapy monitoring at the 6-month follow-up using the same system.

### MR imaging

The CMR protocol included short axis a) CINE and b) LGE images: a) multi-slice 2-dimensional balanced steady-state free-precession CINE with 25 cardiac phases at baseline and follow-up, reconstructed matrix size 174–256 × 208, slices 10–13, and reconstructed voxel size 0.7–1.8 × 0.7–1.8 × 8 mm^3^; b) Phase sensitive inversion recovery T1-weighted gradient-echo LGE sequences acquired after 10 min intravenous administration of 0.2 mmol/kg gadopentetate dimeglumine (Magnograf; Marotrast GmbH, Jena, Germany), with reconstructed matrix size 192 × 144, slices 10–13, and voxel size 1.4–2.1 × 1.4–2.1 × 0.8–3.2 mm^3^.

### PET imaging

At baseline, 40 to 50 min list-mode fluorine-18 fluorodeoxyglucose (FDG) PET data were acquired simultaneously with the MR acquisition. Patient preparation included a one-hour hyperinsulinaemic-euglycaemic clamp procedure, followed by FDG intravenous injection of 333 ± 30 MBq (4 MBq prescribed per kg body weight) an hour prior to the scanning [11]. The PET data were corrected for the dead time, scatter, and attenuation, and then PET images were reconstructed using an ordered-subset-expectation maximization iterative reconstruction algorithm with 3 iterations and 21 subsets (zoom 1), with reconstructed matrix size 344 × 344, 127 slices, and voxel size 2 × 2 × 2 mm^3^. Attenuation correction was based on a 2-point Dixon-based MRI sequence and the maximum-likelihood reconstruction of attenuation and activity algorithm (for arm truncations correction [26]).

### Imaging analysis

The parameters FDG uptake, LGE signal transmurality, and wall motion abnormality (hereafter named FDG, LGE, and WMA) were extracted from the PET, CMR-LGE, and CMR-CINE images, respectively, to evaluate the metabolism, the scar transmurality, and the wall motion of the left ventricle myocardium. These variables were regionally assessed using a 5-point scoring system in the 17-segment American Heart Association myocardial model [27].

At baseline, the FDG and LGE 5-point scores were 0: 100 %, 1: 75–100 %, 2: 50–75 %, 3: 25–50 %, and 4: 0–25 % (with 100 % the maximal uptake per subject), and 0: none, 1: 0–25 %, 2: 25–50 %, 3: 50–75 %, and 4: 75–100 %, respectively. At baseline and follow-up, the WMA 5-point scores were 0: normal wall motion, 1: mild to moderate hypokinesia, 2: severe hypokinesia, 3: akinesia, and 4: dyskinesia [11].

Moreover, the myocardial wall motion recovery (Recovery) was defined as a binary variable representing the WMA improvement between follow-up and baseline in two classes 0: unrecovered or worsening, 1: recovered [11]. Consequently, for each subject, 17 myocardial wall segments were characterized by the FDG, LGE, and WMA scores, and the Recovery classes.

### Machine learning workflow

**Fig. 1:**
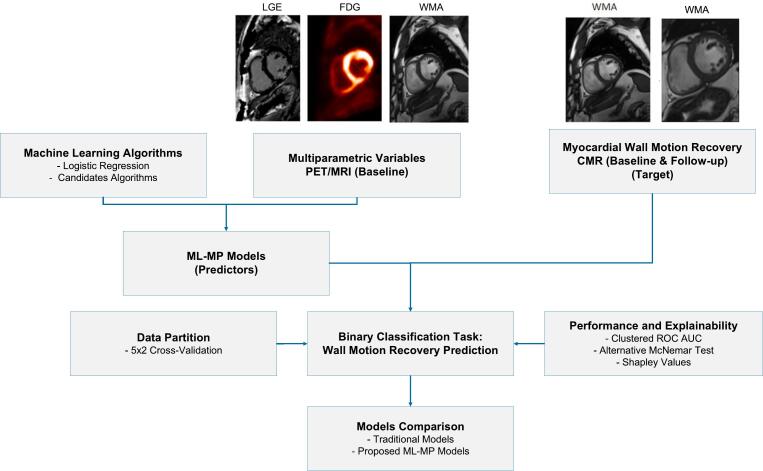
Machine learning workflow. Machine learning workflow for prediction of myocardial wall motion recovery based on FDG-PET/MRI viability assessment examination and CMR therapy monitoring 6 months after CTO revascularization. PET/MRI: positron emission tomography / magnetic resonance imaging, CMR: cardiac magnetic resonance, LGE: late gadolinium enhanced scar transmurality, FDG: Fluorine-18 fluorodeoxyglucose uptake, WMA: wall motion abnormalities, ML-MP models: machine learning multiparametric models, ROC AUC: receiver operating characteristic area under the curve.

Fig. 1 illustrates the machine learning workflow for the binary classification task wall motion recovery prediction. The predictor models were composed by grouping the machine learning algorithms and the PET/MRI multiparametric variables (ML-MP models), while the target was the CMR myocardial wall motion recovery. The implementation considered the guidelines for clinical prediction models to assure transparency and to reduce risks of biased results [[28], [29], [30]]. Moreover, since the dataset contained multiple myocardial segments per subject, intracluster correlations were expected [31], and therefore, clustered matched-pair data considerations were addressed during the machine learning workflow.

### Machine learning algorithms and multiparametric variables

The predictor models were defined by the pair {Algorithm} – {Multiparametric Variables}. The algorithms used in this study were the linear methods logistic regression (LogReg) [32], and logistic regression with mixed effects (MELogReg) [33], and the nonlinear algorithms decision tree (DecTree) [34], k-nearest neighbors (KNN) [35], Naive Bayes (NBayes) [36], random forest (RF) [37], and support vector machine (SVM) [38]. The multiparametric variables used per algorithm were the single FDG, LGE, or WMA, or the combinations LGE + FDG, LGE + WMA, or LGE + FDG + WMA.

These selections were based on the following points: LogReg and FDG, LGE, and LGE + FDG were employed in the previous studies [10,11] and therefore, considered the baseline models in this study; MELogReg is used to consider intra-subject correlation effects [39]; and the extensive list of the well-established nonlinear algorithms [[40], [41], [42]] and the remaining parameters were used to have a pool of alternatives to find patterns that improve the recovery prediction.

### Data partition

The ML-MP models were trained and tested through a cross-validation partition (K-fold CV) to get an estimation of the generalizability of the models’ performance. Moreover, the pipeline was repeated R times to account for sampling variance and model stability (RxK-fold CV) in small datasets [43].

The CV scheme applied was a 2-fold cross-validation with 5 repetitions (5×2 CV). Namely, the full dataset was divided 5 times into two folds, generating 5 sets of folds with different data sampling. Hence, in each repetition the predictor models were trained and tested as usual in a classical CV preserving the machine learning workflow and using the full dataset, but the data points allocated in each fold changed between repetitions. Also, two folds (K = 2) were used to have the largest possible test sets and disjoint training sets, accounting for data variation and avoiding overlapping between folds in both sets and thus reducing the underestimation of the metrics variance. This partition scheme has proven the utility in detecting true differences between algorithms in binary classification tasks [43].

Additionally, all myocardial segments of each subject were assigned to the same fold (grouping by subjects’ ID) to avoid data leakage [44], and each fold was stratified by the recovery classes to preserve recovery distribution. Lastly, the minority events were sampled up by random replication (over-sampling with replacement) to handle class imbalance during the training phase.

### Prediction performance and explainability

The prediction performance was assessed with the metric area under the receiver operating characteristics curve adapted for clustered matched-pair data (cROC AUC) [[45], [46], [47]]. The models’ marginal probabilities of success were compared via an alternative McNemar test, a combined 5x2 CV F-test for clustered matched-pair data. Mathematical development and computational implementation were based on previous works [[48], [49], [50]]. Development details are in the supplemental material.

The prediction explainability per model was computed via the Shapley values. This is a model-agnostic explainable artificial intelligence approach based on the cooperative game theory for feature contribution estimation [[51], [52], [53]]. For each ML-MP model, a Global Shapley value was estimated aggregating the absolute value of the local mean Shapley (the mean Shapley values [52] per variable score in each CV fold) among all scores (0–4) and variables (FDG, LGE, and/or WMA) that the respective model is consisted of, and lastly, among the repetitive CV folds.

### ML-MP models comparison

ML-MP models for wall motion recovery prediction were studied in two groups: 1) comparison of traditional models, and 2) comparison of the proposed models. The reference point was the model LogReg - LGE + FDG (i.e., a classifier based on logistic regression and the variables FDG and LGE) recommended in the earlier works [10,11]:

1. Traditional Models
The performance of the reference model was compared with the models LogReg - FDG and LogReg - LGE. This analysis aimed to assess the reliability and consistency of the reference model:

$$\mathrm{LogReg}-\mathrm{LGE}+\mathrm{FDG}\;\mathrm{vs}\;\left\{\mathrm{LogReg}\right\}-\left\{\mathrm{FDG},\mathrm{LGE}\right\},$$

where {LogReg} − {FDG, LGE} represents the models LogReg − FDG and LogReg - LGE.

2. Proposed ML-MP Models
Performance and explainability were contrasted between the reference model and the ML-MP models conformed by the algorithms LogReg, MELogReg, DecTree, KNN, NBayes, RF, and SVM, and the multiparametric variables FDG, LGE, WMA, LGE + FDG, LGE + WMA, and LGE + FDG + WMA, with the objective of identifying the most effective model for predicting wall motion recovery:

$$\mathrm{LogReg}-\mathrm{LGE}+\mathrm{FDG}\;\mathrm{vs}\;\left\{\mathrm{Algorithms}\right\}-\left\{\mathrm{Multiparametric}\;\mathrm{Variables}\right\}.$$

### Workflow and models validation

The cROC AUC values were calculated in training and testing sets for all the ML-MP models to validate the model generalizability. The gap performance was computed as the difference between the cROC AUC values obtained during training and testing and compared between the ML-MP models.

In addition to the 5×2 CV approach, three auxiliary experiments with 5×3, 5×5 and 5×10 CV were performed to investigate the performance consistency across sampling size variance. These setups corresponded to 50 %, 66.6 %, 80 %, and 90 % of the dataset size used for model training. Moreover, the workflow (100 % dataset used for training, no testing set) and performances from the previous study [11] were retrieved for comparison.

### Statistical analysis

The cROC AUC and Global Absolute Shapley values, along with their respective percentage differences between ML-MP models and the reference model (Diff cROC AUC, Diff Global Absolute Shapley) are presented as mean and standard errors. The statistical tests were adjusted for multiple ML-MP models comparisons using Bonferroni's method. We introduced five criteria to determine whether a ML-MP model differed significantly from the reference model:

1. Diff cROC AUC > 10 % [54]
2. Diff cROC AUC p < .05 (two-tailed Z-tests against zero, with p values combined via Bonferroni’s method [55])
3. F-test p < .05 (5x2 CV F-tests for clustered matched-pair data for the marginal probabilities of success between the ML-MP model and the reference)
4. Diff Global Absolute Shapley > 20 %
5. Diff Global Absolute Shapley p < .05. (two-tailed correlated paired t-tests against zero [56,57])

Furthermore, two extra variables were added to the list of ML-MP models as a sanity check for both workflow and comparison analysis. The fictitious variables “Simulation Perfect Case” and “Simulation Flawed Case” were generated by sampling the target Recovery, matching the 100 %, and the 50 % of the class's events, correspondingly. Therefore, the predictor models built with these variables should theoretically get the best, and an unremarkable performance, respectively.

The data analysis framework was based on the R (version 4.4.1) [32], and the machine learning collection package “tidymodels” [58], with library management handled via “renv” [59]. The corresponding R project is available in [60] with a fictitious dataset.

## Results

### Dataset description and patient characteristics

The final dataset analyzed in this study consisted of 79 myocardial wall segments with a class proportion of 21:58 for recovered:unrecovered wall motion after PCI revascularization. This number represented the segments with WMA > 0 at baseline in the coronary arteries affected by the CTO. The included segments corresponded to 21 subjects (patients characteristics in Table 1), with 11 participants having at least one segment recovered after PCI, while the remaining 10 did not have any wall motion improvement in the follow-up. Consequently, in the context of 5x2 CV, on average the folds used for training and testing included 39 ± 8 segments.

**Table 1:** Demographics. Demographic information of chronic total occlusion cohort used in this study. CAD: coronary arterial disease, CTO: chronic total occlusion. * Missing drug information for one subject.

Demographic Characteristics (n = 21)	
Male Sex (%)	20 (95 %)
Age (years)	62 ± 9
Body mass (kg/m^2^)	29 ± 3
Medical History	
Diabetes	5 (24 %)
Hypertension	19 (90 %)
Smoker (former/current)	12 (57 %)
Medication	angiotensin-converting enzyme 20 (95 %)*
	beta-blockers 19 (90 %)*
	diuretics 9 (43 %)*
	statins 20 (95 %)*
Dyslipidemia	17 (81 %)
Family history	6 (29 %)
Multivessel CAD	19 (90 %)
Previous myocardial infarction	10 (48 %)
Coronary artery bypass	3 (14 %)
Disease Characteristics	
CTO Localisation	LAD 2 (9 %)
	LCX 6 (29 %)
	RCA 13 (62 %)
Ejection Fraction (%)	47 ± 14
End-diastolic volume (ml)	149 ± 39
End-systolic volume (ml)	79 ± 33

### Performance comparison

#### Traditional models

Table 2 presents the performance of the models composed of LGE + FDG, LGE, and FDG using logistic regression. LGE + FDG outperformed FDG (difference −35 (17) %, cROC AUC p < 0.0001), but did not exceed LGE values (difference 11 (12) %, cROC AUC p > 0.05), whose performance was the highest with a cROC AUC of 0.61 (0.07). Moreover, there were no statistically significant differences between the marginal probabilities of success of the three models (F-test p > 0.05).

**Table 2:** Traditional model comparison. Performance comparison in myocardial wall motion recovery prediction between the traditional multiparametric model LGE+FDG and single variables. Values represented with mean values and standard errors. LogReg: logistic regression, LGE: late gadolinium enhanced scar transmurality, FDG: Fluorine-18 fluorodeoxyglucose uptake, cROC AUC: receiver operating characteristics area under the curve for clustered data.

Multiparametric variables	Algorithm	cROCAUC	Diff cROCAUC [%]	Diff cROCAUC p	F-Test p
LGE + FDG	LogReg	0.55 (0.07)	0 (0)		
FDG	LogReg	0.41 (0.08)	−35 (17)	<0.0001	>0.05
LGE	LogReg	0.61 (0.07)	11 (12)	>0.05	>0.05

#### Proposed machine learning – Multiparametric models

**Fig. 2:**
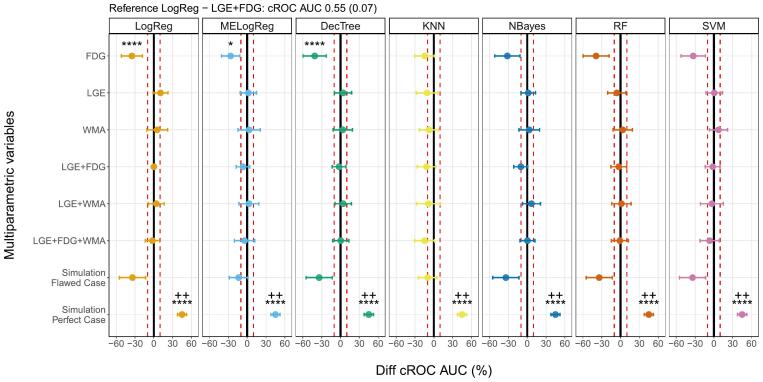
cROC AUC ML-MP comparison. Percentage difference in cROC AUCs between the multiparametric variables with different machine learning algorithms and the reference LogReg - LGE+FDG. The solid lines represent LogReg - LGE+FDG cROC AUC, and the dashed lines the differences ±10 %. *, **, ***, and **** stand for p value < 0.05, p value < 0.01, p value < 0.001, and p value < 0.0001, respectively, from the Z-test used in the cROC AUC comparison between proposed models and the reference. +, ++, +++, ++++ stand for p value < 0.05, p value < 0.01, p value < 0.001, and p value < 0.0001, respectively, from the F-test used in the marginal probabilities of success comparison between proposed models and the reference. LogReg: logistic regression, MELogReg: logistic regression with mixed effects, DecTree: decision tree, KNN: k-nearest neighbor, NBayes: Naive Bayes, RF: random forest, SVM: support vector machine, LGE: late gadolinium enhanced scar transmurality, FDG: Fluorine-18 fluorodeoxyglucose uptake, WMA: wall motion abnormalities, cROC AUC: receiver operating characteristics area under the curve for clustered data.

Fig. 2 presents an overview of the difference between cROC AUCs for all the models and the reference computed in the 5x2 cross-validation approach. There were no significant improvements in cROC AUC with the proposed ML-MP models. No combinations between LGE, FDG and WMA and the classification algorithms presented higher values than 10 % of the LogReg - LGE + FDG model. Simpler standalone LGE and WMA models had comparable values to the reference, while FDG performed the worst with differences lower than 10 %. In addition, there were no statistically significant differences in the marginal probabilities of success between the models and LogReg - LGE + FDG. Looking at the simulated variables, all the models with the perfect case presented statistically significant differences in the cROC AUC values (>30 %) and in the marginal probabilities of success when comparing with the reference model. The flawed case models predominantly showed cROC AUC lower than 10 % of the reference’s value.

#### Explainability comparison

**Fig. 3:**
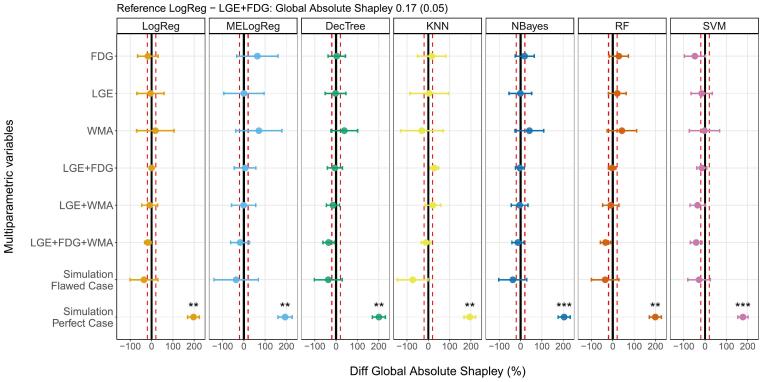
Global Absolute Shapley comparison. Percentage difference in the Global Absolute Shapley between the multiparametric models with different machine learning algorithms and the reference model LogReg - LGE+FDG. The solid lines represent no differences with respect to LogReg - LGE+FDG, and the dashed lines the differences ± 20 %. *, **, ***, and **** stand for p value < 0.05, p value < 0.01, p value < 0.001, and p value < 0.0001, respectively, from the t-test used in the Global Absolute Shapley comparison between proposed models and the reference. LogReg: logistic regression, MELogReg: logistic regression with mixed effects, DecTree: decision tree, KNN: k-nearest neighbor, NBayes: Naive Bayes, RF: random forest, SVM: support vector machine, LGE: late gadolinium enhanced scar transmurality, FDG: Fluorine-18 fluorodeoxyglucose uptake, WMA: wall motion abnormalities.

Fig. 3 depicts the differences between the Global Absolute Shapley values for all the models and the reference. Proposed multiparametric models did not show significant differences among all the algorithms (values within the range ± 20 % of reference LogReg - LGE + FDG 0.17(0.05)). In particular, standalone models presented similar values as the reference and in some cases mean values were higher than 20 % (MeLogReg - WMA and FDG, DecTree - WMA, NBayes - WMA, and RF- WMA). In terms of the simulated variables, all the models with the perfect case presented statistically significant differences in the Global Shapley values (>150 %), while the flawed case models showed mean values lower than the reference’s Global Absolute Shapley (<20 %).

**Fig. 4:**
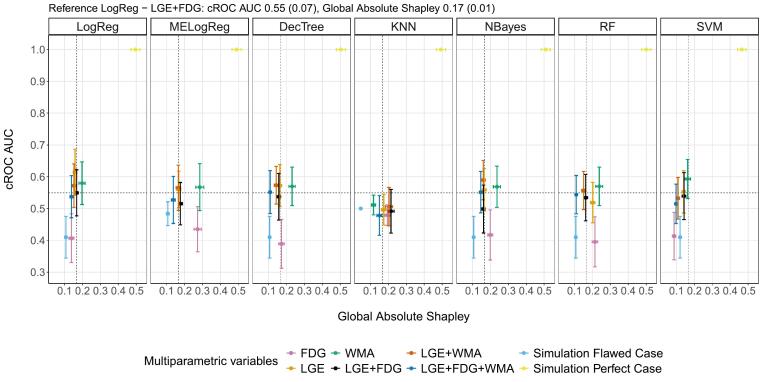
Metrics comparison. Global Absolute Shapley vs cROC AUC per multiparametric models with different machine learning algorithms. The dashed lines represent the values obtained with the reference model LogReg - LGE+FDG. LogReg: logistic regression, MELogReg: logistic regression with mixed effects, DecTree: decision tree, KNN: k-nearest neighbor, NBayes: Naive Bayes, RF: random forest, SVM: support vector machine, LGE: late gadolinium enhanced scar transmurality, FDG: Fluorine-18 fluorodeoxyglucose uptake, WMA: wall motion abnormalities.

Lastly, the combination of the performance and explainability metrics among all ML-MP models is reported in Fig. 4. LogReg - LGE presented the highest mean cROC AUC value, while WMA with most all the algorithms had higher mean cROC AUC value and mean Global Absolute Shapley value than LogReg - LGE + FDG. Also, the perfect case models presented the maximum values in cROC AUC (mean values of 1.0) and Global absolute Shapley values (mean values > 0.4), while the flawed case models had low values (mean values < 0.5 and 0.15, respectively) together with the FDG models.

### Workflow and models validation

#### Generalization assessment

**Fig. 5:**
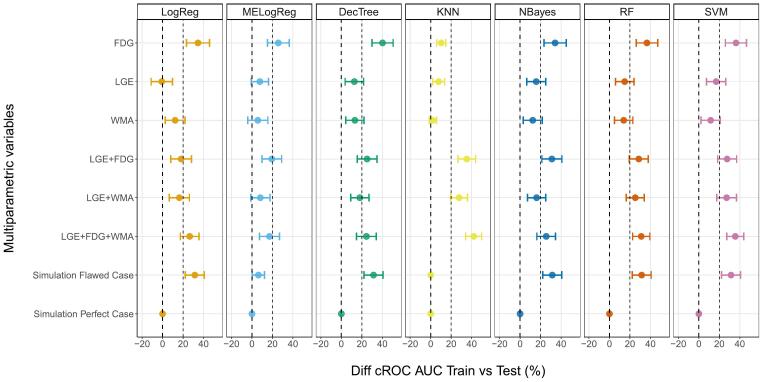
Performance Gap. Percentage difference between cROC AUCs computed in training and testing set for each multiparametric variable and machine learning algorithm. The dashed lines represent the differences 0 % and 20 % between training and testing performance. LogReg: logistic regression, MELogReg: logistic regression with mixed effects, DecTree: decision tree, KNN: k-nearest neighbor, NBayes: Naive Bayes, RF: random forest, SVM: support vector machine, FDG: Fluorine-18 fluorodeoxyglucose uptake, LGE: late gadolinium enhanced scar transmurality, WMA: wall motion abnormalities, cROC AUC: receiver operating characteristics area under the curve for clustered data.

The comparison between the cROC AUCs calculated in training and testing sets for all the ML-MP models (the performance gap) is reported in Fig. 5. In general, the performance gaps among the models were below 45 %. Models with LogReg and MELogReg presented gaps > 20 % only in 2/6 of the proposed MP variables (excluding simulations), while when using RF and SVM the gaps > 20 % were presented in 4/6. The MP variables LGE + FDG + WMA and FDG showed gaps > 20 % in 6/7 algorithms, while LGE and WMA mostly had gaps < 20 %. Also, perfect cases showed no gaps, while the gaps in flawed cases were mostly greater than 20 %.

#### Performance across sample size variance

**Fig. 6:**
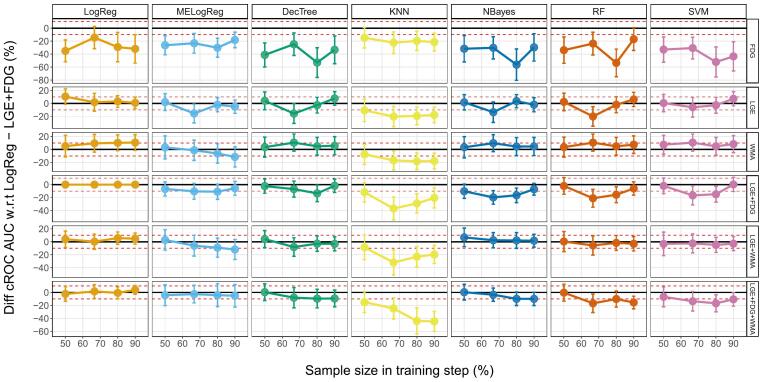
Sampling Variance. Percentage difference in cROC AUCs between the multiparametric variables with different machine learning algorithms and the reference LogReg - LGE+FDG vs sample size used during models training. The solid lines represent no difference with respect to LogReg - LGE+FDG cROC AUC value, and the dashed lines the differences ±10 %. LogReg: logistic regression, MELogReg: logistic regression with mixed effects, DecTree: decision tree, KNN: k-nearest neighbor, NBayes: Naive Bayes, RF: random forest, SVM: support vector machine, FDG: Fluorine-18 fluorodeoxyglucose uptake, LGE: late gadolinium enhanced scar transmurality, WMA: wall motion abnormalities, cROC AUC: receiver operating characteristics area under the curve for clustered data.

Fig. 6 summarizes the performance comparison between the ML-MP models and the reference among different subsample sizes. The Diff cROC AUC values do not exceed 10 % in any of the subsample configurations studied (RxK CVs: 5×2, 5×3, 5×5, 5×10). This shows the consistency of our findings among sampling variance: the lack of improvements with more complex models and the similar performance between the reference and the simpler standalone models.

**Fig. 7:**
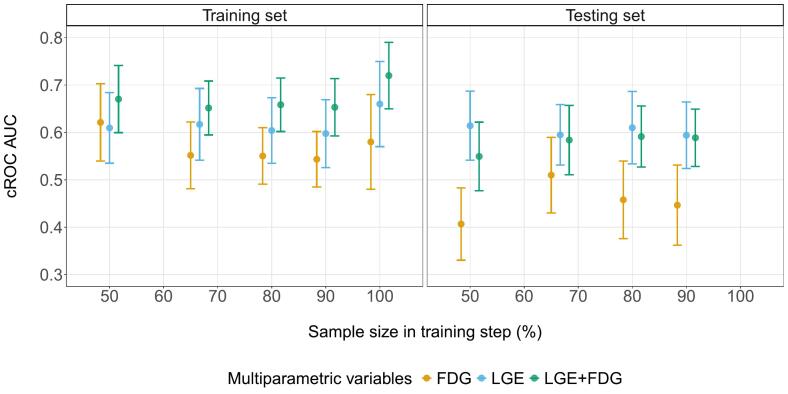
Experiments Comparison. cROC AUCs comparison between traditional multiparametric model LGE+FDG and single variables, computed in training and testing sets from different workflow experiments. FDG: Fluorine-18 fluorodeoxyglucose uptake, LGE: late gadolinium enhanced scar transmurality, cROC AUC: receiver operating characteristics area under the curve for clustered data.

Focusing on the performance of the traditional models, Fig. 7 depicts the comparison between the workflow and the results from this work (50–90 % sample size in training step) and from the previous study (100 % sample size in training step) [11]. Mean values computed in training sets were higher than those computed in testing sets. LGE + FDG presented higher performance than the FDG and LGE for all the approaches during training, however, the superiority against LGE vanished when evaluating in an unseen subset. These results emphasize the over-optimistic performance obtained by the reference model when the comparison is only evaluated in the training set (case 100 %).

## Discussion

This study comprehensively evaluated the potential of FDG-PET/MRI multiparametric analysis for predicting myocardial wall motion recovery after CTO revascularization. Our investigation addressed critical aspects of machine learning classifiers, including generalizability estimation, model explainability, intra-subject correlations, and sampling-derived bias. The results in this cohort revealed the following:

1. Multiparametric PET/MRI analysis combining LGE and FDG using logistic regression was not significantly superior to the single component CMR LGE.
2. Contrary to our hypothesis, incorporating more complex multiparametric PET/MR interactions using FDG, LGE, and/or WMA with nonlinear algorithms did not boost the recovery prediction.
3. Standalone models CMR WMA and CMR LGE may represent simpler and more effective candidates for myocardial recovery prediction.

Specifically, the linear combination of scar transmurality (MRI-LGE) and metabolism (PET-FDG) did not present generalizable synergistic effects when identifying the myocardial segments to recover, where the metabolism information did not improve the prediction. One reason might be a potential collinearity between the two variables [61] which therefore does not provide supplementary information to the models. As a subsequent analysis, in favor of this reasoning we found a moderate association between LGE and FDG (Cramer [62] median value 0.28, and interquartile [0.25, 0.29] in the 5x2 cv experiment).

In comparison with the literature, our results do not support the recommendation from the two closest PET/MRI studies [10,11]. The discrepancies might be a consequence of the cross-validation partition scheme that implies conducting the training in smaller sets, and thereby impacting the algorithm performances. However, all our cross-validation experiments (including the approach with sample size similar to the previous study [11]) showed lower cROC AUC values and unremarkable differences between the models. Then, our robustness assessment highlights the importance of validation steps to avoid overoptimistic results [63]. It is essential to recall that the aim of the myocardial recovery prediction is the assistance in the risk stratification before an invasive procedure, and thus robust workflows with generalizable results are crucial in clinical settings.

Furthermore, the proposed more complex multiparametric models did not present significant benefits in recovery prediction. As mentioned with the traditional comparison, collinearity between the features may impact the model’s performance. In this case, moderate to strong associations were found between the three variables (Cramer median value 0.39, and interquartile [0.38, 0.42] between the pair LGE and WMA, Cramer median value 0.30, and interquartile [0.22, 0.31] between FDG and WMA). The risk of overfitting might be also a cause for discrepancy in some models. The nonlinear algorithms RF and SVM showed a performance gap greater than 20 %, together with the multiparametric model including all variables. This illustrates the need for further studies with larger sample sizes to fully explore the capacities of nonlinear algorithms. Nevertheless, the performance across different K-folds experiments still showed that no more complex model performed better than the linear reference, that is, the lack of improvements is robust to sample variance (under the small CTO cohort limitation). Lastly, potential performance improvements might be reached by tuning the algorithms’ hyperparameter, however, this involves splitting the small partition folds into even smaller internal validation sets. To note, the nonlinear combinations of FDG and LGE did not enhance the prediction, showing that the traditional multiparametric variables already saturated their potential with a simpler algorithm. In alignment to this results, a meta-analytic study highlighted the lack of machine-learning-based improvements in similar medical applications [29]. They claimed that machine learning did not outperform logistic regression models in clinical prediction tasks, and remarked that papers with overoptimistic reports used workflows with high risks of biased results [29,30].

Additionally, these results revealed that no multiparametric models challenged the single-modalities LGE or WMA in performance and explainability. This raises the central question whether multiparametric FDG-PET/MRI analysis really enhances the prediction of myocardial wall motion recovery after revascularization. Going even further, the highest cROC AUC value 0.61 (0.07) among all the models was indeed much closer to the flawed classifier than to the perfect classifier. This underscores the need for new concepts beyond the oversimplified binary viability [9,64], whose main hypothesis has already been challenged by PCI trials results [[65], [66], [67]]; and for more comprehensive prediction models in patient management strategy [5,68,69].

### Limitations and future directions

Some limitations of this study are the sample size and the class imbalance of the dataset: the small number of patients exhibited a small number of CTO subtended myocardial segments with abnormal wall motion and the majority of those did not recover after six months of perfusion restoration. Even though these drawbacks were addressed here with 1) the implementation of a workflow via an iterative k-fold cross-validation for sampling bias consideration, and 2) class balancing the training dataset; studies with larger sample sizes are necessary to further support these results.

Cross-validation partitions are the optimal option for estimating the generalizability of a model’s performance in small datasets [43]. However, this approach presents the risk of variance bias, since training and testing steps are performed in subsets (with reduced data distribution representativeness), in place of using the full set with the real data distribution [70]. Therefore, external validation datasets are still needed for more accurate and realistic generalizability estimations.

In terms of the multiparametric assessments, perfusion imaging could improve the models’ performance [7,[71], [72], [73]], along with the incorporation of clinical data [74,75]. Also, the semi-quantitative scores used as predictors and outcomes (LGE, FDG, and WMA) are potentially sensitive to inter-observer variability and may not detect intermediate conditions between scores. Hence, thorough automatic-quantitative assessment should be considered in future works [76,77].

Finally, can multiparametric FDG-PET/MRI analysis really enhance the prediction of myocardial recovery after CTO revascularization? Our results suggest reviewing the utility of this multiparametric approach. Still, to pursue generalizable clinical evidence, new investigations should include larger and more extensive patient populations in terms of sex and age, more diverse clinical condition severity, further follow-up points, multi-scanner/site data, and with multiparametric variables and multimodal data beyond combinations of FDG, LGE, and WMA. Moreover, these future studies are encouraged to follow the guidelines in clinical prediction tasks (PROBAST, CHARMS, TRIPOD, CLAIM, etc.) [28,[78], [79], [80], [81]] to build robust workflows and thus to avoid overoptimistic results [62].

## Conclusion

This study evaluated FDG-PET/MRI multiparametric models for predicting regional functionality recovery following perfusion restoration, addressing key challenges such as generalizability estimation, model explainability, intra-subject correlations, and sampling-derived bias.

The evaluation revealed that linear and nonlinear interactions incorporating multiparametric data, including cardiac function, infarct extension, and metabolism offered no significant predictive advantages for myocardial wall motion recovery after revascularization in a small CTO cohort. Despite the application of machine learning algorithms, the benefits of multiparametric FDG-PET/MRI analysis for risk stratification in PCI-revascularization remain inconclusive.

To strengthen these findings, further studies should focus on larger cohorts, external validation datasets, and workflows with low risk of bias. Moreover, the development of novel models incorporating advanced multiparametric cardiac imaging and multimodal clinical data beyond infarct extension and metabolism, is encouraged to evaluate innovative approaches for pre-PCI risk stratification.

## CRediT authorship contribution statement

**Alberto Villagran Asiares:** Writing – original draft, Validation, Software, Methodology, Formal analysis, Data curation, Conceptualization. **Teresa Vitadello:** Writing – review & editing, Resources, Project administration, Methodology, Data curation. **Osvaldo M. Velarde:** Writing – review & editing, Validation, Methodology, Formal analysis. **Sylvia Schachoff:** Writing – review & editing, Investigation. **Tareq Ibrahim:** Writing – review & editing, Resources. **Stephan G. Nekolla:** Writing – review & editing, Supervision, Resources, Funding acquisition, Conceptualization.

## Funding

This work was part of a project that has received funding from the European Union’s Horizon 2020 research and innovation program under the Marie Skłodowska-Curie Grant Agreement No 764458.

## Declaration of competing interest

The authors declare that they have no known competing financial interests or personal relationships that could have appeared to influence the work reported in this paper.
