# Supplementary material for: Can multiparametric FDG-PET/MRI analysis really enhance the prediction of myocardial recovery after CTO revascularization? A machine learning study
Source: Z Med Phys. 2025 Apr 23;36(1):84–98. doi: 10.1016/j.zemedi.2025.03.003 (PMC12901531; doi:10.1016/j.zemedi.2025.03.003)
Supplement: Supplementary Data 1 [file mmc1.docx]

Supplemental Material

### Section A: Mathematical Scheme and Implementation Details of the Combined 5x2 F Test for Clustered Matched-Pair Data

The marginal probabilities of success between two models were compared by an alternative of McNemar test, that accounts for the correlation between the segments from the same subjects [1], and the CV approach [2]: combined 5x2CV F test for clustered matched-pair data.

### A.1 Combined 5x2 CV F-Test:

Alpaydin [2] proposed an f statistic that follows an F distribution with 10 and 5 degrees of freedom to compare the marginal probabilities of success of the two models in a 5x2 cross-validation experiment:

$f=\frac{\sum_{i=1}^{R=5} \sum_{j=1}^{J=2} \left( p_{i}^{\left( j \right)} \right)^{2}}{2*\sum_{i=1}^{R=5} \left( s_{i} \right)^{2}}$,

where $p_{i}^{\left( j \right)}$ is the difference between the marginal probabilities of success of the two classifiers $p_{a}$ and $p_{b}$ on fold $j$ = 1, 2 on replication $i$ = 1, …, 5:

$p_{i}^{\left( j \right)}=\left( p_{a}-p_{b} \right)_{i}^{\left( j \right)} \left( 1 \right)$.

Then, considering the average and estimated variance across the two folds on the replication $i$:

$${p‾}_{i}=\frac{p_{i}^{\left( 1 \right)}+p_{i}^{\left( 2 \right)}}{2} \left( 2 \right)$$

$\left( s_{i} \right)^{2}=\left( p_{i}^{\left( 1 \right)}-{p‾}_{i} \right)^{2}+\left( p_{i}^{\left( 2 \right)}-{p‾}_{i} \right)^{2} \left( 3 \right)$,

and replacing [*Equation 2*](#kix.kuzeevudnrmf) in [*Equation 3*](#kix.3bfmz3wbqvvt):

$$\left( s_{i} \right)^{2}=\left( p_{i}^{\left( 1 \right)}-\frac{p_{i}^{\left( 1 \right)}+p_{i}^{\left( 2 \right)}}{2} \right)^{2}+\left( p_{i}^{\left( 2 \right)}-\frac{p_{i}^{\left( 1 \right)}+p_{i}^{\left( 2 \right)}}{2} \right)^{2}=$$

$$\left( s_{i} \right)^{2}=\left( \frac{p_{i}^{\left( 1 \right)}-p_{i}^{\left( 2 \right)}}{2} \right)^{2}+\left( \frac{-p_{i}^{\left( 1 \right)}+p_{i}^{\left( 2 \right)}}{2} \right)^{2}=$$

$$\left( s_{i} \right)^{2}=2\left( \frac{p_{i}^{\left( 1 \right)}-p_{i}^{\left( 2 \right)}}{2} \right)^{2}$$

$\left( s_{i} \right)^{2}=\frac{\left( p_{i}^{\left( 1 \right)}-p_{i}^{\left( 2 \right)} \right)^{2}}{2} \left( 4 \right)$,

the f statistic can be formulated as:

$$f=\frac{\sum_{i=1}^{R=5} \sum_{j=1}^{J=2} \left( p_{i}^{\left( j \right)} \right)^{2}}{2*\sum_{i=1}^{R=5} \frac{\left( p_{i}^{\left( 1 \right)}-p_{i}^{\left( 2 \right)} \right)^{2}}{2}}$$

$$f=\frac{\sum_{i=1}^{R=5} \sum_{j=1}^{J=2} \left( p_{i}^{\left( j \right)} \right)^{2}}{\sum_{i=1}^{R=5} \left( p_{i}^{\left( 1 \right)}-p_{i}^{\left( 2 \right)} \right)^{2}}$$

$f=\frac{\sum_{i=1}^{R=5} \left( p_{i}^{\left( 1 \right)} \right)^{2}+\left( p_{i}^{\left( 2 \right)} \right)^{2}}{\sum_{i=1}^{R=5} \left( p_{i}^{\left( 1 \right)}-p_{i}^{\left( 2 \right)} \right)^{2}} \left( 5 \right)$.

### A.2 Clustered Matched-Pair Data,:

Durkalski [1] presented a mathematical framework to compute the marginal probabilities of success of the classifier models in the situation of clustered data. Considering a prediction task based on a dataset with $N$ Total number of observations, $K$ number of subjects (clusters), and $n_{k}$ number of observations in subject $k$, the corresponding contingency table $C$ between two predictor models A and B is:

|  | Model B  (Standard / Reference) |  |  |
| --- | --- | --- | --- |
| **Model A** | Success | Failure | Total |
| Success | $\sum_{k=1}^{K} a_{k}$ | $\sum_{k=1}^{K} b_{k}$ | $x_{ak}=\sum_{k=1}^{K} \left( a_{k}+b_{k} \right)$ |
| Failure | $\sum_{k=1}^{K} c_{k}$ | $\sum_{k=1}^{K} d_{k}$ |  |
| Total | $x_{bk}=\sum_{k=1}^{K} \left( a_{k}+c_{k} \right)$ |  | $\sum_{k=1}^{K} n_{k}=N$ |

The overall marginal probabilities of success for both models can be estimated as:

$p_{a}=\frac{1}{K}\sum_{k=1}^{K} \frac{x_{ak}}{n_{k}}=\frac{1}{K}\sum_{k=1}^{K} \frac{a_{k}+b_{k}}{n_{k}}$,

$p_{b}=\frac{1}{K}\sum_{k=1}^{K} \frac{x_{bk}}{n_{k}}=\frac{1}{K}\sum_{k=1}^{K} \frac{a_{k}+c_{k}}{n_{k}}$,

and then, the difference between the probabilities as:

$$p=\left( p_{a}-p_{b} \right)=\frac{1}{K}\sum_{k=1}^{K} \frac{a_{k}+b_{k}}{n_{k}}-\frac{1}{K}\sum_{k=1}^{K} \frac{a_{k}+c_{k}}{n_{k}}$$

$$p=\frac{1}{K}\sum_{k=1}^{K} \frac{b_{k}-c_{k}}{n_{k}} \left( 6 \right)$$

In the context of a cross-validation experiment of $j$’s folds and $i$’s repetitions, from each corresponding contingency table $C_{i}^{\left( j \right)}$ the marginal probabilities difference between the models A and B can be extended from [*Equation 6*](#kix.aw7ifepnsww9):

$p_{i}^{\left( j \right)}=\frac{1}{K_{i}^{\left( j \right)}}\sum_{k=1}^{K_{i}^{\left( j \right)}} \left( \frac{b_{k}-c_{k}}{n_{k}} \right)_{i}^{\left( j \right)} \left( 7 \right)$.

### A.3 Combined 5x2 F Test for Clustered Matched-Pair Data:

Considering a clustered dataset with $K$ clusters, in order to compare the marginal probabilities of success of two classification models a and b in a 5x2 cross-validation experiment, and thus to determine whether they statistically differ each other, the combined 5x2 F test for clustered matched-pair data can be estimated using the f statistic from [*Equation 5*](#kix.1b9tpeddz8fo):

$f=\frac{\sum_{i=1}^{R=5} \left( p_{i}^{\left( 1 \right)} \right)^{2}+\left( p_{i}^{\left( 2 \right)} \right)^{2}}{\sum_{i=1}^{R=5} \left( p_{i}^{\left( 1 \right)}-p_{i}^{\left( 2 \right)} \right)^{2}}$,

and the marginal probabilities differences from [*Equation 7*](#kix.2pif1tlxeebe):

$p_{i}^{\left( 1 \right)}=\frac{1}{K_{i}^{\left( 1 \right)}}\sum_{k=1}^{K_{i}^{\left( 1 \right)}} \left( \frac{b_{k}-c_{k}}{n_{k}} \right)_{i}^{\left( 1 \right)} \left( 8 \right)$,

$p_{i}^{\left( 2 \right)}=\frac{1}{K_{i}^{\left( 2 \right)}}\sum_{k=1}^{K_{i}^{\left( 2 \right)}} \left( \frac{b_{k}-c_{k}}{n_{k}} \right)_{i}^{\left( 2 \right)} \left( 9 \right)$,

$p_{i}^{\left( 1 \right)}-p_{i}^{\left( 2 \right)}=\frac{1}{K_{i}^{\left( 1 \right)}}\sum_{k=1}^{K_{i}^{\left( 1 \right)}} \left( \frac{b_{k}-c_{k}}{n_{k}} \right)_{i}^{\left( 1 \right)}-\frac{1}{K_{i}^{\left( 2 \right)}}\sum_{k=1}^{K_{i}^{\left( 2 \right)}} \left( \frac{b_{k}-c_{k}}{n_{k}} \right)_{i}^{\left( 2 \right)} \left( 10 \right)$.

Lastly, [*Equation 5*](#kix.1b9tpeddz8fo), [*Equation 8*](#kix.445x37l9fkn8), [*Equation 9*](#kix.fk6zdso85fgs), and [*Equation 10*](#kix.jokavbmm2afi) were implemented by adapting functions of the R package ‘clust.bin.pair’ [3] (adaptations available with the R framework of this study). The combined 5x2CV F test for clustered matched-pair data was evaluated on the two simulated models against the reference LogReg - LGE + FDG. Thus, under the hypothesis that “the performance of the latter is better than a random classifier but still not the highest”, it was expected to have statistically significant differences in the marginal probabilities of success between the “perfect” and “flawed” cases against the reference. F-test p-values are displayed in Figure A.

| 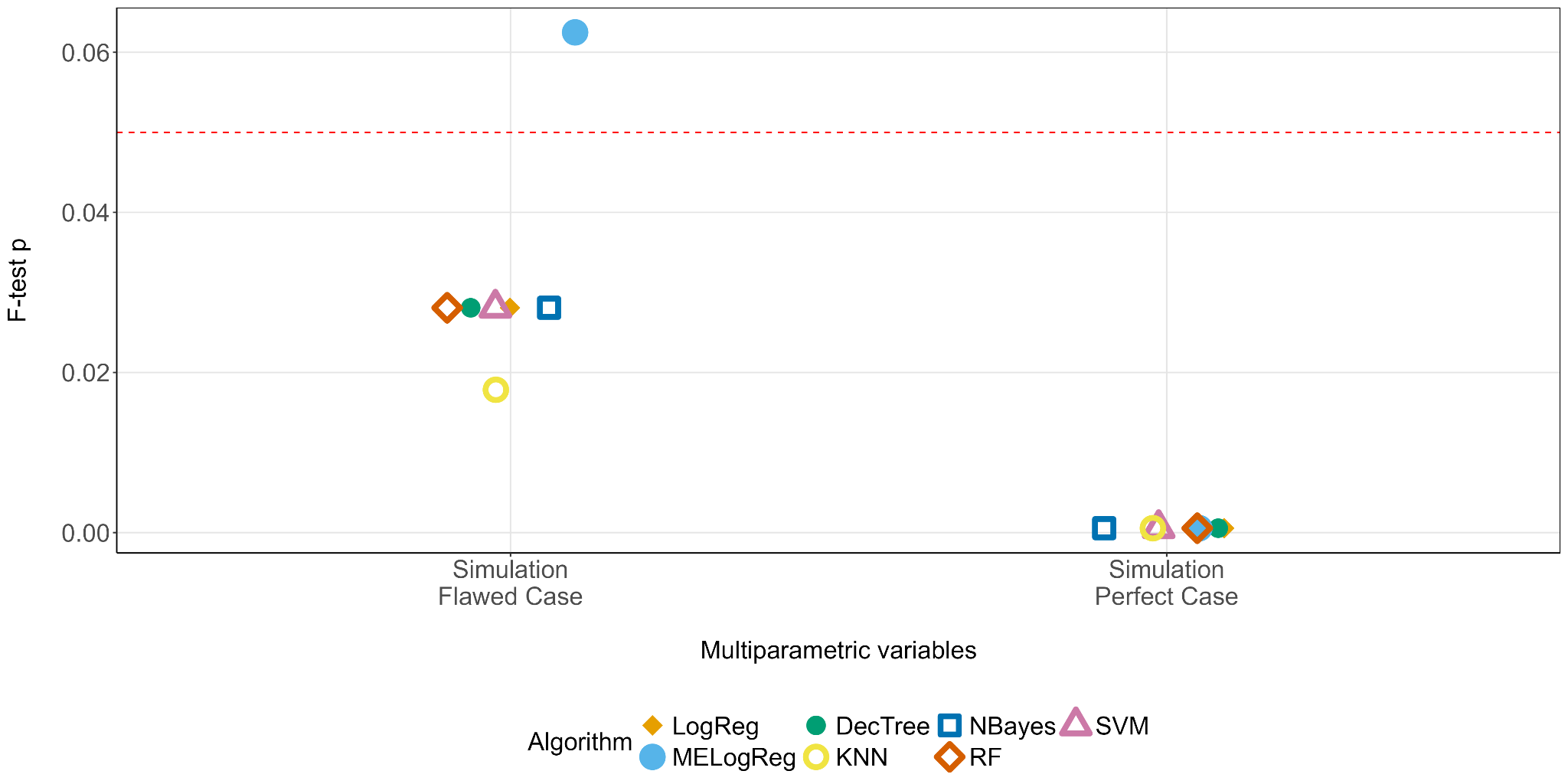 |
| --- |
| Figure A. F-test simulations  Comparison between simulated models and the reference model LogReg - LGE + FDG using the combined 5x2CV F-test for clustered matched-pair data. P-values not corrected for multiple comparisons. The dashed line represents p-value = 0.05. LogReg: logistic regression, MELogReg: logistic regression with mixed effects, RF: random forest, SVM: support vector machine, KNN: k-nearest neighbor. |

References in Supplementary Material

[1] Durkalski VL, Palesch YY, Lipsitz SR, Rust PF. Analysis of clustered matched-pair data. Stat Med. 2003;22(15):2417-28.

[2] Alpaydin E. Combined 5 x 2 cv F test for comparing supervised classification learning algorithms. Neural Comput. 1999;11(8):1885-92.

[3] Gopstein D. clust.bin.pair: Statistical Methods for Analyzing Clustered Matched Pair Data. 2018.
